# Sparse-view tomography via displacement function interpolation

**DOI:** 10.1186/s42492-019-0024-7

**Published:** 2019-11-12

**Authors:** Gengsheng L. Zeng

**Affiliations:** 1 Department of Engineering, Utah Valley University, 800 West University Parkway, Orem, UT 84058 USA; 2 Department of Radiology and Imaging Sciences, University of Utah, 729 Arapeen Drive, Salt Lake City, UT 84108 USA

**Keywords:** Limited data imaging, Tomography, Estimation

## Abstract

Sparse-view tomography has many applications such as in low-dose computed tomography (CT). Using under-sampled data, a perfect image is not expected. The goal of this paper is to obtain a tomographic image that is better than the naïve filtered backprojection (FBP) reconstruction that uses linear interpolation to complete the measurements. This paper proposes a method to estimate the un-measured projections by displacement function interpolation. Displacement function estimation is a non-linear procedure and the linear interpolation is performed on the displacement function (instead of, on the sinogram itself). As a result, the estimated measurements are not the linear transformation of the measured data. The proposed method is compared with the linear interpolation methods, and the proposed method shows superior performance.

## Introduction

Low-dose computed tomography (CT) can be achieved by using either lower current or fewer projection views. Sparse-view tomography thus can find applications in low-dose CT [1]. Another application is in fast magnetic resonant imaging (MRI) when radial k-space sampling scheme is utilized [2]. In sparse-view tomography, measurements are under-sampled, which usually result in severe aliasing artifacts as streaking lines in the reconstructed images. The phrase “sparse-view” means that the number of views in data acquisition is significantly smaller than the value that is met by Shannon’s sampling theorem, which requires [3].

1 $$ {N}_p\approx {D}_{min} $$

where *N*_*p*_ is the number of views over 180° and *D*_*min*_ is the minimum number of detector bins to cover the object at one view. Many systematic studies lead to more efficient sampling criteria [4–8], where more complicated two-dimensional interpolations are discussed.

According to compressed sensing theory, sparse-view tomography may still be possible if some image domain constraints are used to compensate for the missing data [9–15]. However, this paper focuses on analytic filtered backprojection (FBP) reconstruction. Iterative reconstruction methods and machine learning methods are beyond the scope of this paper.

Sometimes the unmeasured views can be approximately estimated by interpolation methods [16–19]. Recently, machine learning methods are popular and successful in sparse-view data estimation [20–22].

In this paper, we argue that interpolation with linear convolution approaches simply introduce slightly rotated images to the main image. By the “main” image, we imply the image that is reconstructed by only the measured sparse data. The results of machine learning methods depend on the training sets; therefore, the results may not be applicable to all applications. Here we propose a nonlinear method to estimate unmeasured projections by using displacement functions, which will be discussed in Section II. The linear interpolation method assumes that the unmeasured value is the average of its neighbors. On the other hand, the deformation method assumes that the unmeasured value has the same value as one of its neighbor’s value. The deformation method is nonlinear and is able to avoid or reduce the rotational interpolation artifacts. Comparison simulations will be presented in Section III, and Section IV concludes the paper.

## Methods

### The rotation effect of the linear interpolation method

As a simple example to illustrate the main motivation, this paper first considers a naïve linear interpolation approach that doubles the number of views in tomography. Let the sinogram be *p*(*n*, *m*), where *p* is the line integral of the object, *n* is the detector bin index and *m* is the view angle index. In this example, when *m* is odd, the *p*(*n*, *m*) is measured. When *m* is even, the *p*(*n*, *m*) is not measured and needs to be estimated. A simple linear interpolation scheme to estimate *p*(*n*, 2 *m*) from *p*(*n*, 2 *m*-1) and *p*(*n*, 2 *m* + 1) is

2 $$ p\left(n,2m\right)=0.5\times \left[p\left(n,2m-1\right)+p\left(n,2m+1\right)\right] $$

**Fig. 1:**
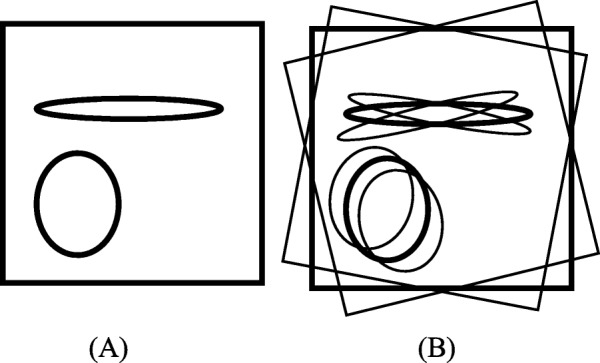
Outline diagrams for images reconstructed (**a**) from the original under-sampled sinogram and (**b**) from interpolated sinogram

The ultimate effect of this interpolation scheme is exaggeratingly illustrated in Fig. 1 as an outline drawing, where the under-sampling streaking artifacts are not shown. Figure 1a shows the main image reconstructed from the original sinogram, while Fig. 1b shows the image reconstructed from the interpolated sinogram using (2). It is interesting to observe from Fig. 1b that the reconstructed image from the interpolated sinogram is a combination of three components: the main reconstruction using the original under-sampled sinogram (with a weighting factor of 1), a rotated version of the main reconstruction by Δγ (with a weighting factor of 0.5), and a rotated version of the main reconstruction by -Δγ (with a weighting factor of 0.5). Here 2Δγ is the angular gap between two adjacent views in the original under-sampled sinogram.

An intuitive way to understand this phenomenon is to consider a different example that copies the available measurements at view 2 *m* + 1 to view 2 *m*, i.e., *p*(*n*, 2*m*) = *p*(*n*, 2*m* + 1). Using the extended sinogram, the reconstructed image will be the summation of two images: one is the original image and the other is a rotated version of the original image.

In general, sinogram interpolation via convolution yields an image that is a combination of the main reconstruction and some rotated versions of the main reconstruction. Similar phenomena are expected for other sinogram estimation methods that based on linear interpolation.

These two simple examples imply that in order to significantly improve the sinogram estimation, we must use some sort of nonlinearity. The idea of non-rigid deformation may be borrowed, altered, and applied to our sinogram estimation [23–25]. Another non-linear way is to use sine wave approximation [17]. This paper proposes a displacement function interpolation method.

### Use the deformation function for non-linear interpolation

The main idea of our algorithm is illustrated below. A pair of measured sinogram views is provided: *p*(*n, m*_1_) and *p*(*n, m*_2_), where *n* is the index along the radial direction and *m*_1_ and *m*_2_ are two angular indices. The goal is to estimate *p*(*n, m*) with *m* between *m*_1_ and *m*_2_.

The first step of the proposed method is to find a displacement function *u*(*n*) to connect *p*(*n, m*_1_) and *p*(*n, m*_2_) so that

3 $$ p\left(n,{m}_2\right)\approx p\left(n+u(n),{m}_1\right) $$

It is desired to find a displacement function *u*(*n*) by minimizing the objective function

4 $$ {F}_n=\left[p\left(n,{m}_2\right)-p\right(n+n(u),{m}_1\Big]{}^2 $$

for each *n*. Since *n* is an index, we could require *n*(*u*) to be an integer.

It is fairly flexible how to define an objective function *F*. As another example, we can add the sign function of the finite difference to the objective function as.

$$ F={\left[p\left(n,{m}_2\right)-p\left(n+u(n),{m}_1\right)\right]}^2 $$

5 $$ +\lambda \left[\operatorname{sign}\left(p\left(n,{m}_2\right)-p\left(n-1,{m}_2\right)\right)-\mathit{\operatorname{sign}}\Big(p\left(n+u(n),{m}_1\right)-p\left(n+u(n)-1,{m}_1\right)\right)\Big]{}^2 $$

where λ is a pre-set parameter to balance the weighting between constraints in the objective function *F*. We set λ = 0.01 in our implementation of (5). The purpose of the sign function is to encourage that the slopes of the deformed function *p*(*n* + *u*(*n*), *m*_1_) and the target function *p*(*n*, *m*_2_) have the same sign.

If we restrict *u*(*n*) to be integers in [−*N*, *N*] with *N* being a pre-set positive integer, it is efficient to evaluate the objection *F* with all possible *u*(*n*) values in [−*N*, *N*] and use a “*min*” function to determine the optimal displacement function *u*(*n*). Here, “*min*” is a built-in function in Matlab® to find the minimum value in an array. The motivation of using the “*min*” function instead of an iterative algorithm (such as the gradient decent algorithm) is to make the algorithm more efficient. The “*min*” function method only evaluates the deformed function 2 *N* + 1 times, while an iterative method evaluates the deformed function at least equal to the number of iterations, which is much greater than 2 *N* + 1.

After the displacement function *u*(*n*) is found, in the second step, the un-measured sinogram *p*(*n, m*) with *m* between *m*_1_ and *m*_2_ can be readily obtained by linearly interpolating the displacement function *u*(*n*). For example, if *m*_2_ – *m*_1_ = *M* + 1, we can estimate *M* views between *m*_1_ and *m*_2_ as

6 $$ p\left(n,m\right)\approx p\left(n+\frac{m-{m}_1}{M}u(n),{m}_1\right) $$

for *m* = *m*_1_, *m*_1_ + 1, …, *m*_2_ − 1. We must point out that in (6) *n* + *u*(*n*) × (*m* − *m*_1_)/*M* is most likely not an integer. Let

7 $$ {n}_1=\left\lfloor n+\frac{m-{m}_1}{M}u(n)\right\rfloor $$

and

8 $$ \alpha =n+\frac{m-{m}_1}{M}u(n)-{n}_1 $$

where ⌊*x*⌋ is the largest integer that is not greater than *x*. Then (6) cab be expressed as

9 $$ p\left(n,m\right)\approx \left(1-\alpha \right)p\left({n}_1,{m}_1\right)+\alpha p\left({n}_1+1,{m}_1\right) $$

for *m* = *m*_1_, *m*_1_ + 1, …, *m*_2_ − 1.

**Fig. 2:**
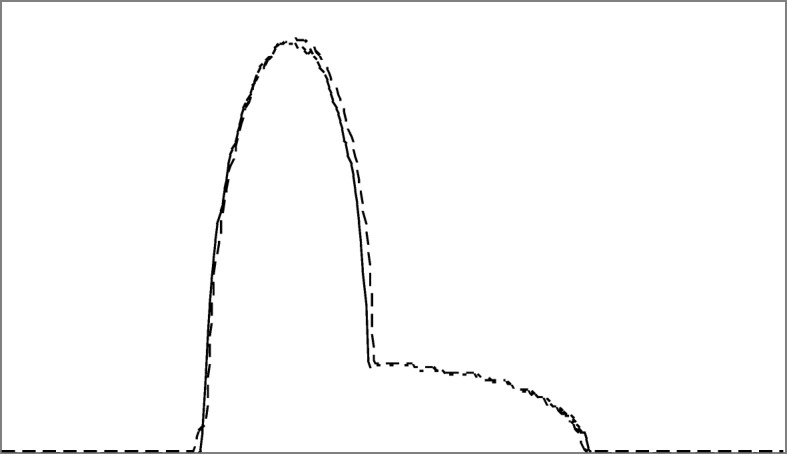
Projections at two adjacent view angles from the original under-sampled sinogram. One is shown as a broken curve. The other is shown as a solid curve

**Fig. 3:**
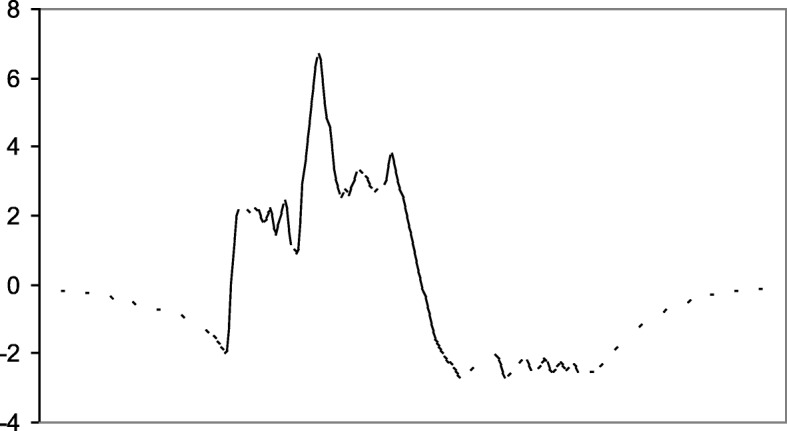
A typical displacement function, *u*(*n*), that deforms projection values from one measured view to its adjacent measured view in the original under-sampled sinogram. The vertical axis represents the displacement, *u* (*n*). The horizontal axis represents the pixel indices, *n*

An illustrative example for the proposed estimation procedure is shown in Fig. 2, where we have two sinogram measurements: *p*(*n*, 2 *m* + 1), as a broken curve, and *p*(*n*, 2 *m*-1), as a solid curve. Our proposed algorithm discussed above gives a displacement function, *u*(*n*), as shown in Fig. 3, so that

10 $$ p\left(n,2m+1\right)\approx p\left(n+u(n),2m-1\right) $$

The unmeasured *p*(*n*,2 *m*) is then estimated as

11 $$ p\left(n,2m\right)\approx p\left(n+0.5\times u(n),2m-1\right) $$

Since 0.5*u*(*n*) may not be an integer, linear interpolation is required as suggested by (9). Similarly, *p*(*n*, 2*m*) can be estimated by the measurement at view 2 *m* + 1 with a displacement function *v*(*n*), or by the combined measurements at both view 2 *m*-1 and view 2 *m* + 1 as

12 $$ p\left(n,2m\right)\approx \frac{p\left(n+0.5\times u(n),2m-1\right)+p\left(n+0.5\times v(n),2m+1\right)}{2} $$

### Computer simulations and patient study

This proposed sinogram extension method was applied to two computer simulation cases and one real patient case. In the computer simulations, the image size was 256 × 256, and the detector size was 367. In this paper, the word “sinogram” is used in a general sense, and the “sinogram” can be parallel projections and can be fan-beam projections or in other geometries.

In computer simulations, the original under-sampled sinogram was generated analytically without noise. We can better observe the image distortion in noiseless studies. In the first computer simulation study, the original measured number of views was 60 over 360°. After sinogram extension, the number of views was increased to 180 over 360°. In the second computer simulation study, the original measured number of views was 120 over 360°. After sinogram extension, the number of views was increased to 360 over 360°. The absolute error image between the estimated sinogram and the true sinogram was calculated and reported in the next section.

In the patient study, the sinogram data was obtained by a CT scan using 500 mAs. The detector was curved, and the imaging geometry was cone-beam. The central slice of the cone-beam data was used as the fan-beam data. The data set had 896 detector bins at one view and 1200 views over 360°.

In this paper, an under-sampled data set was a subset of the original data set by using only 400 fan-beam views over 360°. After sinogram extension with displacement interpolation, there were 1200 views over 360°.

Sinogram estimation results using two-adjacent-view linear interpolation was also obtained and reported in the next section.

The root mean square error (RMSE) between the reconstruction and the true image was calculated for all reconstruction images. The RMSE is defined as

13 $$ RMSE=\sqrt{\frac{1}{n}\sum \limits_{i=1}^n{\left({R}_i-{T}_i\right)}^2} $$

where *R*_*i*_ is the reconstruction pixel value and *Ti* is the true image value. For the patient study, the true image was not available and is substituted by the reconstruction with the full data set using 1200 views.

For the comparison purposes, an iterative Landweber algorithm was also used in image reconstruction [26]. The iterative Landweber algorithm can be expressed as

14 $$ {X}^{\left(k+1\right)}={X}^{(k)}+\alpha {A}^T\left(A{X}^{(k)}-P\right) $$

where *A* is the projection matrix, *A*^*T*^ is the backprojection matrix, *α* is a relaxation parameter (or step size), *P* is the projection sinogram re-formatted in the vector form, and *X*^(*k*)^ is the reconstructed image at the kth iteration re-formatted in the vector form. The parameter *α* in this paper is chosen as 0.01.

## Results

### Rotation displacement artifacts due to sinogram linear interpolation

Linear interpolation between sinogram views is equivalent to linear combination of the images from the original sparse-view reconstruction and rotated versions of the sparse-view reconstruction. These effects are illustrated by an exaggerated sketch in Fig. 1. The rotational artifacts become more severe at locations away from the center-of-rotation in the image. The observation of these artifacts motivated the investigation of a nonlinear sinogram interpolation method.

### Using function deformation for sinogram interpolation

Figure 2 shows two curves *p*(*n, m*_1_) and *p*(*n, m*_2_), one being a solid curve and the other being a broken curve. These two curves represent two sinogram measurements at view indices *m*_1_ and *m*_2_. A displacement function *u*(*n*) was estimate according to (3) so that the deformed version of one function approximately equal the other function (*p*(*n*, *m*_2_) ≈ *p*(*n* + *u*(*n*), *m*_1_)) . The displacement function is shown in Fig. 3.

Using the displacement function *u*(*n*) for sinogram interpolation was realized as follows. A missing view at the angle exactly between the two measured views can be estimated by replacing *u*(*n*) by 0.5 × *u*(*n*).

### Computer simulations

**Fig. 4:**
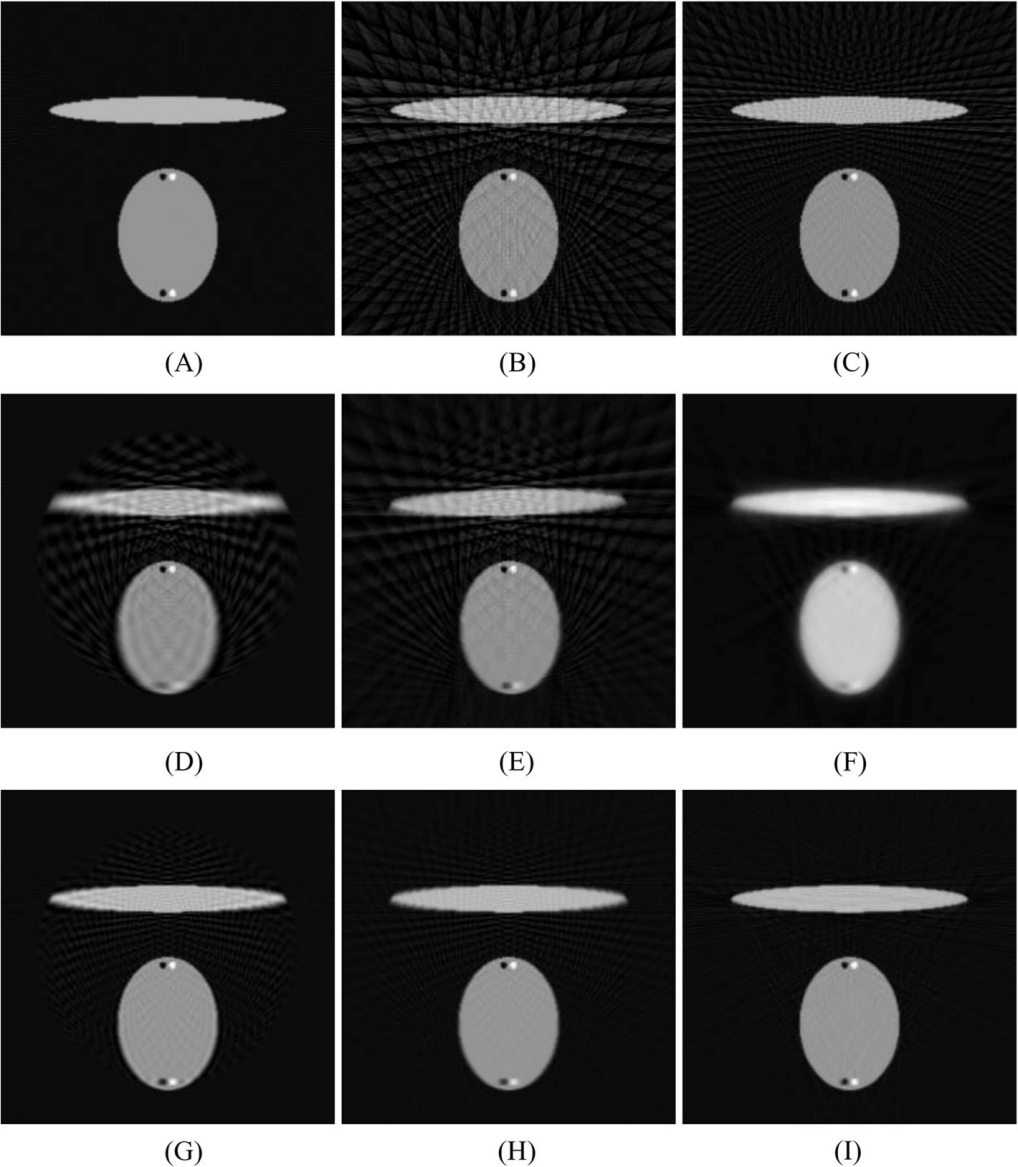
FBP reconstructions reconstructed by **a** 360 measured views, **b** 60 measured views, **c** 120 measured views, **d** 360 views created from 60 views by sinc function interpolation, **e** 360 views created from 60 views by linear interpolation, **f** 360 views created from 60 views by proposed deformation method, **g** 360 views created from 120 views by sinc function interpolation, **h** 360 views created from 120 views by linear interpolation, and **i** 360 views created from 120 views by proposed deformation method

Figure 4 shows the results from the computer simulations with the FBP reconstruction algorithm. In this set, measurements from 360 views over 360° are considered as a full sinogram, and measurements from 60 views over 360° and 120 views over 360° are considered as under-sampled. Figure 4a, b and c show the FBP reconstruction results from the full and under-sampled sinograms, respectively. Fig. 4d, e and f show the results with linear convolution sinogram interpolation methods: sinc function interpolation and linear interpolation, as well as the proposed deformation method, respectively; the initial data set had 60 views. Figure 4g, h and i show the results with linear convolution sinogram interpolation methods: sinc function interpolation and linear interpolation, as well as the proposed deformation method, respectively; the initial data set had 120 views. The linear interpolation method is equivalent to the triangle function convolution method. Figure 4f and i show the results of the proposed non-linear method.

There are two pairs of small black-and-white dots in the phantom. The pair at the bottom is blurred more than the pair at the center be the estimation algorithms. We also observe that for the linear methods there is a circular region and the background noise texture is different within and outside this region.

**Fig. 5:**
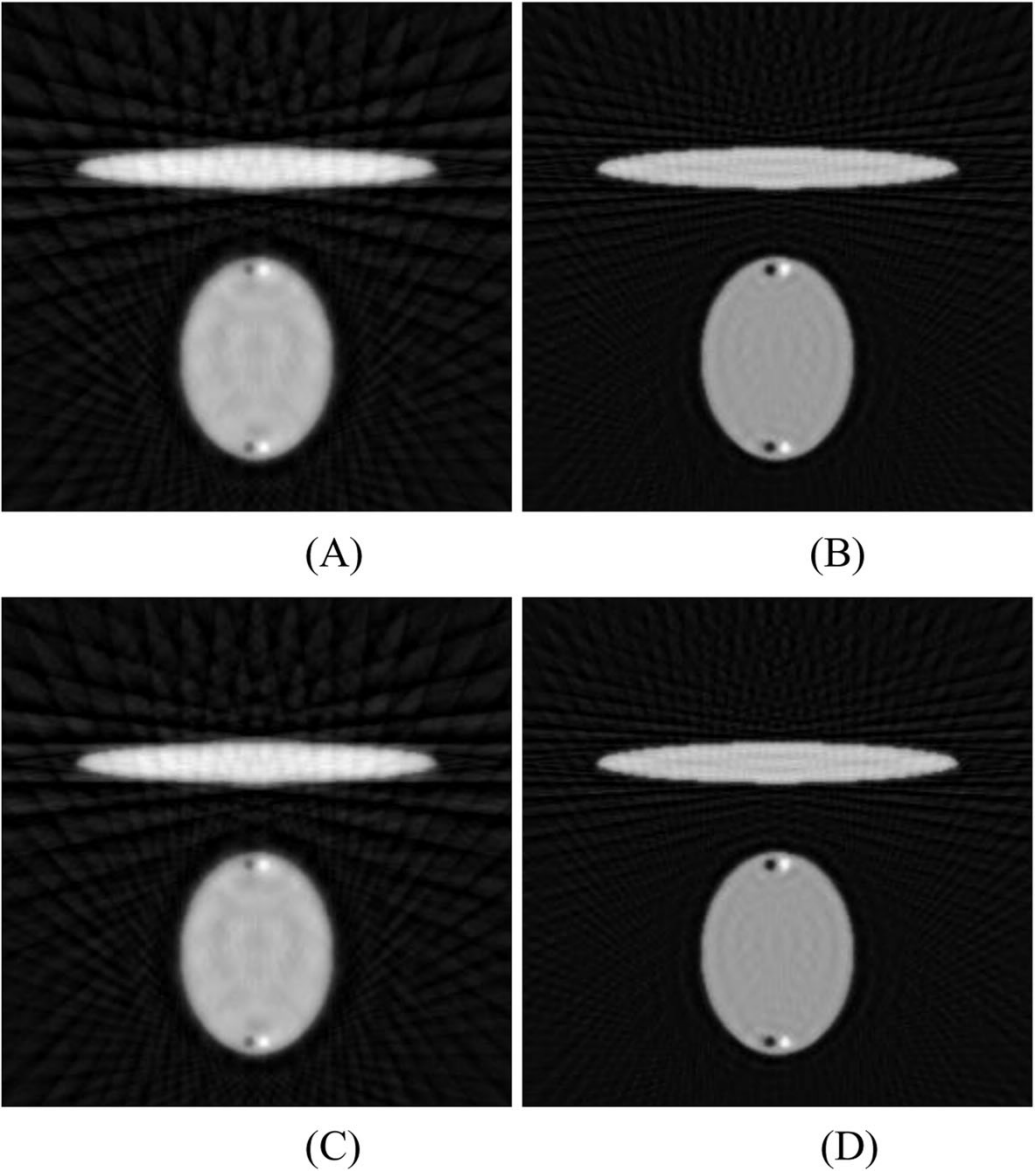
Iterative Landweber reconstructions with 1000 iterations reconstructed by **a** 60 measured views, **b** 120 measured views. Iterative Landweber reconstructions with 2000 iterations reconstructed by **c** 60 measured views, **d** 120 measured views

The iterative Lanweber algorithm was used to reconstruct the image using under-sampled data. The reconstruction results are shown in Fig. 5 for the data set with 60 views and the data set with 120 views, respectively.

**Fig. 6:**
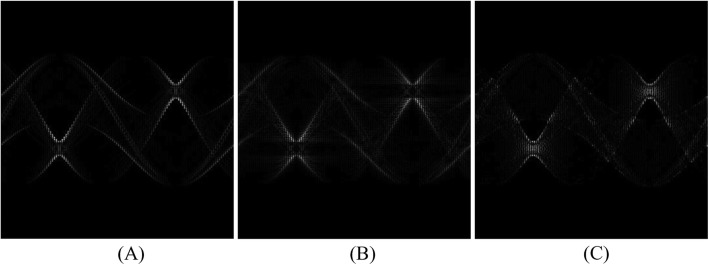
Absolute error between the estimated sinogram and the true sinogram, using **a** linear interpolation method, **b** sinc function interpolation method, and **c** non-rigid deformation method. The original data sets have 120 views and the estimated data sets have 360 views

The estimated sinograms and the true sinogram are compared in terms of the absolute value of the difference in Fig. 6 for the estimation methods used in Fig. 4. A summary of the absolute errors in the estimated sinograms is listed in Table 1. A summary of the RMSE in the FBP reconstructions is listed in Table 2. Table 3 lists the RMSE in the iterative reconstructions. The reconstruction errors for the iterative algorithm reconstructions depend on the iteration number, which is chosen by the user according to the applications. A lower iteration number gives a blurrier image, but less streaking artifacts.

**Table 1:** Computer simulation sinogram estimation errors

Initial data set	Methods	Maximal absolute errors in the expanded sinogram	Sum of absolute errors in the expanded sinogram
60 views	Linear interpolation	0.8635	6419.9
	Sinc interpolation	0.1698	390.2537
	Proposed	0.1254	268.4655
120 views	Linear interpolation	0.1015	108.0924
	Sinc interpolation	0.0898	142.4612
	Proposed	0.0776	97.0789

**Table 2:** Computer simulation FBP reconstruction errors in RMSE

Initial data set	Methods	RMSE
60 views	Raw under-sampled data	0.0612
	Linear interpolation	0.0640
	Sinc interpolation	0.0536
	Proposed	0.0385
120 views	Raw under-sampled data	0.0407
	Linear interpolation	0.0354
	Sinc interpolation	0.0351
	Proposed	0.0282

**Table 3:** Computer simulation iterative reconstruction errors in RMSE

Sinogram views	Number of iterations	RMSE
60 views	1000	0.0364
	2000	0.0362
120 views	1000	0.0681
	2000	0.0680

There are two types artifacts: the under-sampling streaking texture in the uniform areas and the blurry artifacts due to sinogram interpolation. The blurring artifacts can be easily detected by the pair of black-and-white dots at the bottom of the image. All methods perform poorly for the data set that has only 60 views.

**Fig. 7:**
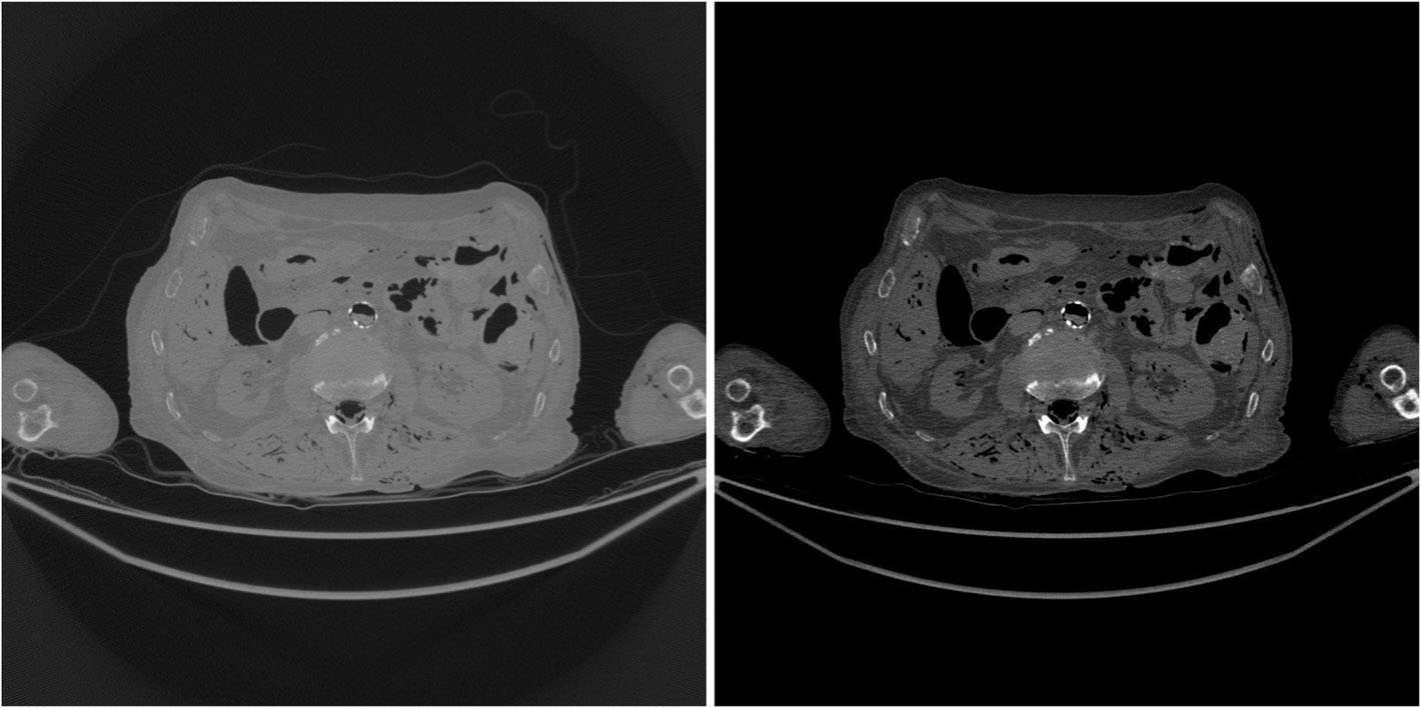
(Gold standard) FBP reconstruction using full sinogram with angular gap = 0.3° (1200 views over 360°). Left image display window: [min, max]. Right image display window: [− 400, 400] HU

**Fig. 8:**
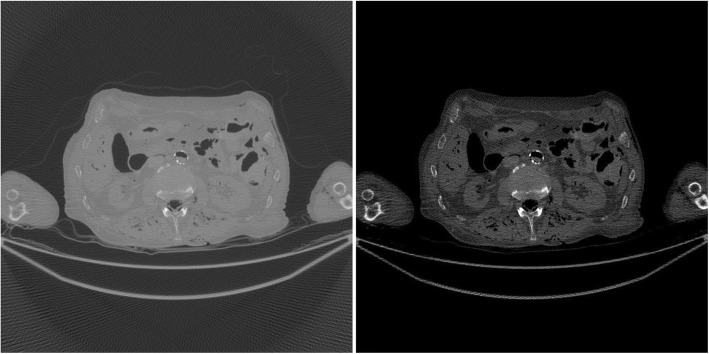
FBP reconstruction using original under-sampled sinogram with angular gap = 0.9° (400 views over 360°). Left image display window: [min, max]. Right image display window: [− 400, 400] HU

**Fig. 9:**
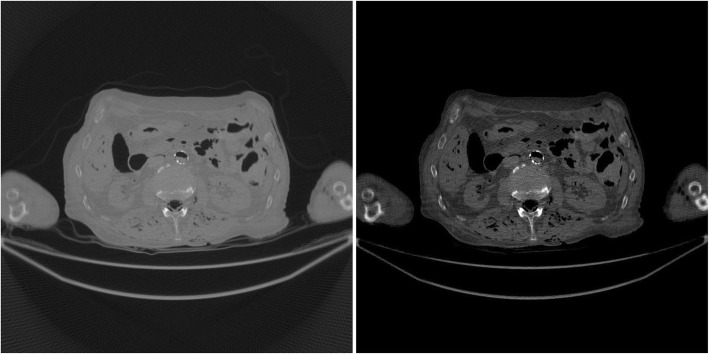
FBP reconstruction using linearly interpolated sinogram with old angular gap = 0.9° and new angular gap = 0.3° (1200 views over 360°). Some rotation artifacts are observed. Left image display window: [min, max]. Right image display window: [− 400, 400] HU

**Fig. 10:**
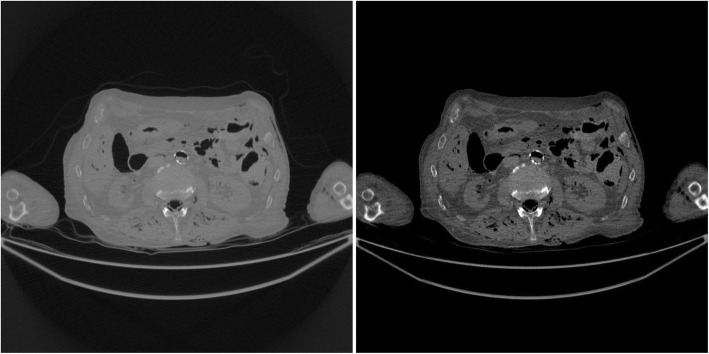
FBP reconstruction using non-rigid deformation interpolated sinogram with old angular gap = 0.9° and new angular gap = 0.3° (1200 views over 360°). This result does not suffer from the rotation artifacts. Left image display window: [min, max]. Right image display window: [− 400, 400] HU

**Fig. 11:**
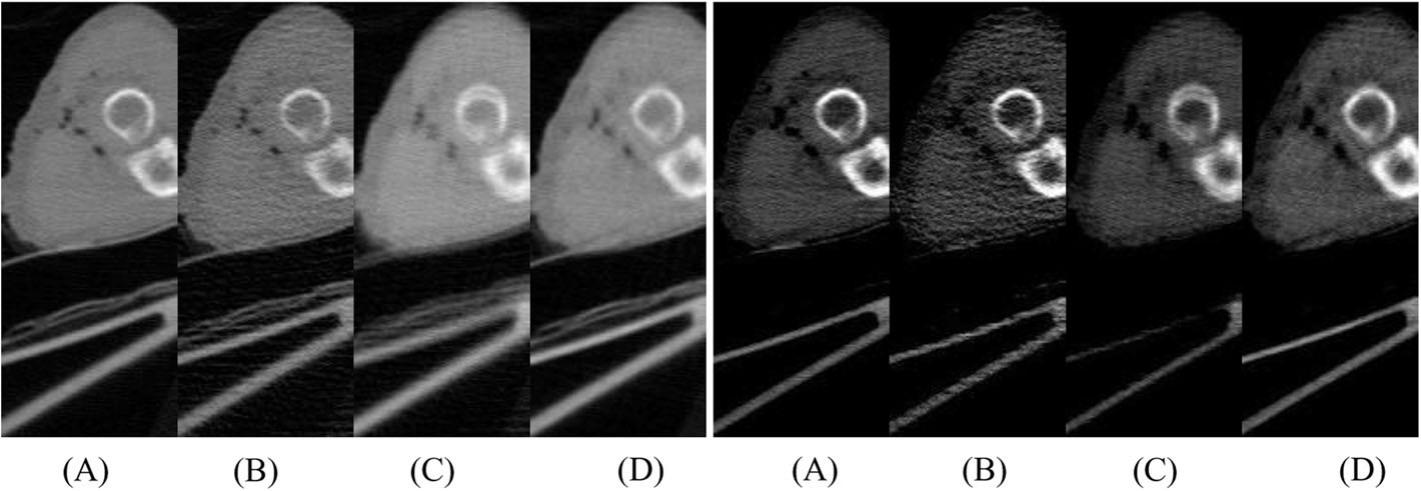
Zoom-in images of a sub rectangular region at the right of each image in Figs. 8, 9, 10, 11 from **a** to **d**. Left images display window: [min, max]. Right images display window: [− 400, 400] HU. Rotation artifacts can be clearly seen in the third column. **a** Using full sinogram (Gold standard). **b** Using sparse sinogram. **c** Using linear interpolation. **d** Using proposed method

**Fig. 12:**
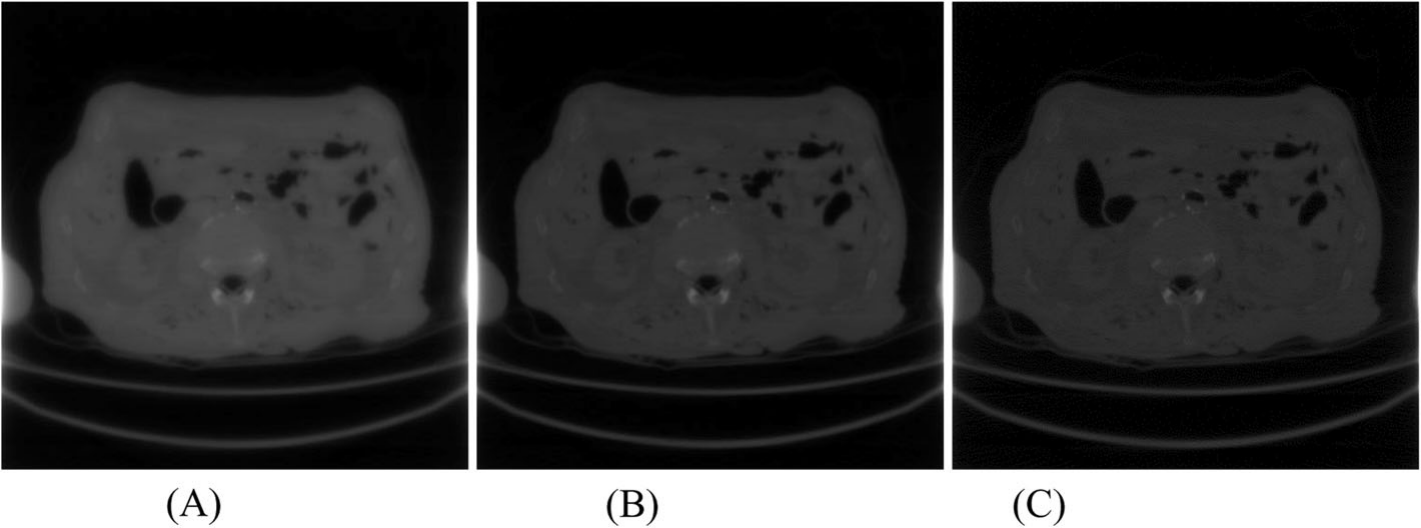
Iterative Landweber algorithm reconstruction using 400 views with iteration number of **a** 500, **b** 1000, and **c** 1500, respectively. The images are still blurry at 1500 iterations

The study results using the patient CT data are shown in Figs. 7, 8, 9, 10, 11, 12. In this study set, measurements from 1200 views over 360° are considered as a full sinogram, and measurements from 400 views over 360° are considered as an under-sampled sinogram. The detector had 896 bins for each view. The reconstructed image size was 800 × 800. Figure 7 shows the FBP reconstruction with this 1200-view full data set and is considered to be the gold standard for other reconstructions to compare with.

Figure 8 shows the FBP reconstruction with 400 views. This image contains lots of streaking artifacts due to angular aliasing. For patient images, all images are displayed twice using two different display windows: [min, max] and [− 400, 400] Hounsfield units (HU).

Figure 9 shows the FBP reconstruction result from linear interpolation method. Severe rotation artifacts are observed in the image. The most severe rotation artifacts are observed at the outer regions inside the patient.

Figure 10 shows the result of proposed method that uses a non-rigid deformation technique. The rotation artifacts are no longer present. However, this image is not perfect. Compared with the gold standard shown in Fig. 7, some shadow artifacts are observed along the high contrast boundaries, and the spatial resolution is somewhat degraded.

In order to appreciate the improvements of the proposed method, a small rectangular sub region at the right part of the original image is cut out and is displayed in a larger format in Fig. 11 for images in Figs. 7, 8, 9, 10.

Figure 12 show three iterative reconstruction images obtained with 500, 1000, and 1500 iterations, respectively. The number of views was 400 over 360°. The image resolution improves as the number of iteration increases. At the 1500th iteration, the reconstructed image is still blurry. RMSEs for the iterative reconstrction results are presented in Table 4 for the patient study.

**Table 4:** Patient study iterative reconstruction errors in RMSE

Sinogram views	Number of iterations	RMSE
400 views	500	0.2705
	1000	0.2703
	1500	0.2703

## Conclusions

Few-view tomography in CT is an open problem. This paper made an observation that linear convolution-based sinogram interpolation methods may produce rotational artifacts. To overcome this problem, this paper suggests a nonlinear method to estimate the unmeasured views. In this proposed method, two adjacent views in the original under-sampled sinogram are used to estimate the missing views between them. A displacement function is estimated by a non-iterative method. A fraction of the displacement function is used to estimate the missing views between the original measurements. One advantage of the proposed method is that the resultant FBP reconstruction using the estimated sinogram does not have the rotation artifacts. Our estimated sinogram is more accurate than the sinogram estimated by linear convolution-based methods, which is demonstrated by the absolution errors as shown in Tables 1, 2 and 3.

In our patient study, there are 400 views over 360° and there are 896 bins on the detector. The number of view angles is extremely small, about 1/4.5 of the value required by the Shannon’s sampling theorem. The proposed algorithm produces fewer artifacts than the linear interpolation method as demonstrated in Fig. 11.

The iterative Lanweber algorithm is also used for the under-sampled data image reconstruction. However, it requires a large number of iterations to produce high resolution images. At the 1500th iteration, the reconstructed image is still blurry.

When the number of views is extremely low, as in the computer simulation with 60 views, the proposed algorithm is not effective, and the reconstructed image is rather blurry even though the streaking artifacts are significantly reduced. It is still an open problem to effectively reconstruct an image with extremely under-sampled data. 

## Data Availability

Not applicable

## Abbreviations

CT: Computed tomography
FBP: Filtered backprojection
HU: Hounsfield units
